# Optimization of Thixotropic Slurry Ratio and Drag Reduction Effect Test for Circular Pipe-Jacking Construction in Pebble Stratum

**DOI:** 10.3390/ma19061148

**Published:** 2026-03-16

**Authors:** Yongzhi Wang, Rui Chen, Anming Wang, Wenli Chen, Zeyu Ren, Xiaogen Li, Pinghui Liu

**Affiliations:** 1 Henan Qianping Reservoir Irrigation District Engineering Co., Ltd., Luoyang 471234, China; 2 College of Geosciences and Engineering, North China University of Water Resources and Electric Power, Zhengzhou 450046, China

**Keywords:** pebble stratum, pipe-jacking construction, thixotropic slurry, drag reduction effect, shell powder

## Abstract

**Highlights:**

**What are the main findings?**

- The optimal thixotropic slurry materials and  mix proportions  (including bentonite, shell powder, etc.) applicable to pipe-jacking construction in pebble strata were established.
- The porous structure of shell powder significantly enhances the slurry’s sealing performance and stratum stability.
- The optimized slurry can reduce the soil friction coefficient by about 35.6%, effectively addressing the issue of high jacking resistance.

**What are the implications of the main findings?**

- An economical and efficient slurry optimization scheme is proposed, utilizing conventional materials such as shell powder, facilitating widespread application.
- It simultaneously addresses challenges in pebble–gravel strata, including high friction, slurry leakage, and stratum instability.
- It provides a successful case for the design of “site-specific slurry” in complex strata, promoting the development of refined construction technologies.

**Abstract:**

Circular pipe-jacking construction in gravel strata faces significant technical challenges, including high frictional resistance, elevated permeability, and susceptibility to collapse. Optimizing the formulation of thixotropic slurry is crucial for improving the construction quality and efficiency of such projects. This study, based on the Ruyang Water Supply Project of the North Main Canal in the Qianping Irrigation Area, Henan Province, China, systematically investigated slurry formulation using bentonite, soda ash, sodium carboxymethyl cellulose (CMC), polyacrylamide (PAM), and shell powder as raw materials. An orthogonal experimental design was employed to optimize the mix proportions, and the friction-reduction performance was validated through drag-friction model tests. The results indicate that the optimal slurry formulation is: bentonite 8%, soda ash 0.3%, CMC 0.2%, PAM 0.15%, shell powder 4%, and water 87.35%. This formulation exhibits excellent fluidity and thixotropy, facilitating the formation of a stable slurry film. Consequently, the friction coefficient between concrete specimens and gravel soil was reduced by 35.6%. The inclusion of shell powder significantly enhanced the slurry’s cohesiveness and improved the anti-seepage capacity of the surrounding stratum due to its filling effect. The optimized thixotropic slurry effectively mitigates frictional resistance during pipe jacking in gravel strata and enhances the formation’s resistance to collapse. The findings of this study provide a viable technical reference for pipe-jacking projects under similar geological conditions.

## 1. Introduction

With the continuous advancement of water conservancy infrastructure in China, trenchless pipe-jacking technology has been increasingly applied in water conveyance pipeline projects. Compared with conventional open-cut methods, this technique reduces ground settlement, minimizes impacts on surrounding structures, and shortens construction periods. As a result, it demonstrates significant technical and economic advantages in long-distance water transmission pipelines traversing urban densely populated areas, major transportation routes, rivers and geologically complex regions. Bentonite slurry is a critical material in pipe-jacking operations, requiring simultaneous possession of favorable lubricity and friction reduction, effective ground support and reliable anti-seepage capacity. These properties are essential to ensure uniform force distribution along the pipeline, reduce frictional resistance, maintain face stability and prevent ground collapse or slurry leakage. Hence, optimizing the mix design and performance of bentonite slurry tailored to various geological conditions represents one of the core challenges in enhancing the safety and efficiency of pipe-jacking construction.

Currently, both domestic and international research on bentonite slurries for pipe jacking under ordinary ground conditions—such as sandy soils [1,2], dry sand layers [3], water-rich sand layers [4], silty clays [5,6], soft clay formations [7], and limestone strata [8]—has yielded numerous mix designs and process guidelines. These studies have provided reliable technical references for conventional pipe-jacking applications. However, under complex geological conditions, particularly in gravel or cobble-rich formations, the loose structure and high permeability of such strata can easily lead to collapse, slurry loss, and increased frictional resistance. These issues contribute to elevated jacking forces, pipeline deviation, or even pipe jamming. Conventional bentonite slurries often fail to meet engineering demands due to their insufficient ability to fill the intergranular voids of gravel matrices and form a robust filter cake, resulting in seepage losses and void formation [9]. Therefore, enhancing slurry performance to improve pore-filling capacity and ensure the stability of the filter cake has become a crucial technical challenge in pipe jacking through gravel-dominated ground.

For pipe jacking in conventional ground conditions, typical slurry constituents include bentonite, soda ash, sodium carboxymethyl cellulose (CMC), and polyacrylamide (PAM), among others. When encountering gravel–cobble strata, researchers often modify these standard mixes by incorporating specialized additives to enhance performance. For instance, in a water-rich cobble layer encountered during a drainage system upgrade project in Kunming, polymer-based chemical slurry powder was introduced into the optimized traditional bentonite mix [10]. This addition significantly improved slurry properties, achieving an approximately 20% increase in friction reduction. During the Yellow River crossing pipe-jacking project—part of the West–East Natural Gas Transmission Pipeline—in a water-rich sandy cobble formation, a powdered integrated lubricant (Bios EXCEED), specifically designed for sandy ground, was employed in slurry preparation [11]. This material effectively mitigated slurry infiltration into the surrounding soil while providing excellent lubrication. In a complex gravel–cobble stratum encountered during a pipeline project for the Zhengzhou New District Wastewater Treatment Plant, a suitable slurry mix was developed using bentonite, soda ash, anionic polyacrylamide (PAM), carboxymethyl starch (CMS), and water [12]. The resulting slurry successfully formed a friction-reducing filter cake, reducing jacking resistance. Furthermore, Wang Chunting et al. [13] conducted a study focusing on slurry lubricity, friction reduction and wall stabilization in gravel–cobble formations. Using raw materials such as bentonite, soda ash, acrylamide, potassium humate and graphite powder, they applied orthogonal experimental design to derive an optimal slurry formulation suitable for large-diameter and long-distance pipe-jacking applications.

It is undeniable that these specialized materials have played a significant role in enhancing slurry performance and reducing friction, with demonstrably positive outcomes. However, their higher cost and more complex handling procedures compared to conventional thixotropic slurry materials may limit their widespread adoption in pipe-jacking projects [14]. Scholars have pointed out that a future trend lies in establishing performance evaluation criteria and standards for thixotropic slurries used in pipe jacking under varying geological conditions and construction specifications [14]. The development of slurry mix designs tailored to different ground conditions remains a major research focus in pipe-jacking technology [15,16,17]. Particularly for pipe jacking in water-rich cobble strata, further experimentation with alternative additives is recommended [10].

Against this background, this study is based on the Ruyang Water Supply Project, North Main Pipe of the Qianping Irrigation District in Henan Province. To address the specific engineering characteristics of cobble strata, we utilized shell powder, which possesses a unique porous microstructure and has been shown to significantly improve slurry water retention, stability, and anti-seepage properties [18]. The research employs bentonite, soda ash, CMC, polyacrylamide (PAM,) and shell powder as raw materials for the thixotropic slurry. Through laboratory orthogonal experiments, the optimal mix proportion were selected. Subsequently, pull-through friction model tests simulating cobble strata were conducted to investigate the practical friction-reduction effectiveness of the developed thixotropic slurry.

## 2. Project Overview

The long-distance pipe-jacking project is located in Ruyang County, along the Beigan Canal of the Qianping Irrigation District in Henan Province, China. The main construction scope involves the development of a new canal-head structure. The pipeline crossing beneath the Beiru River employs a jacking-purpose prestressed concrete cylinder pipe (JPCCP), installed as a single line with a diameter of DN2400. Each pipe segment has a length of 3.0 m. The formation lithology at the construction site primarily consists of gravel strata. A slurry-balance pipe-jacking machine was adopted for installation, which was manufactured by China Railway Engineering Equipment Group Co., Ltd., Henan, China. The muck produced during slurry-balanced jacking is transported via a fully automated slurry pumping system. Lubrication and friction reduction during jacking are achieved using bentonite slurry.

## 3. Mix Proportion Testing of Bentonite Slurry

### 3.1. Performance Indicators of Bentonite Slurry

In various pipe-jacking engineering practices, the focus on the performance of bentonite slurry varies significantly due to differences in geological conditions and construction requirements, leading to a diversified system of evaluation criteria. Zhang Xue et al. [3] proposed key performance indicators for slurry mix proportions in sandy strata for pipe-jacking construction. This study focuses on pipe jacking in gravel strata. Given that both gravel and sandy layers are non-cohesive soils with high permeability, the key performance indicator system for bentonite slurry is established based on the research by Zhang Xue et al. [3], as follows:

(1) Water loss: the volume of filtrate expelled from a certain volume of slurry under a pressure of 0.69 MPa. After water loss, solid particles in the slurry adhere to form a “filter cake”. In pipe-jacking construction, the water loss of bentonite slurry should be ≤25 cm^3^/30 min, with a dense and intact filter cake [19].
(2) Water separation rate: the ratio of the volume of water separated from the slurry after standing for 24 h to the original slurry volume. In construction, the water separation rate of bentonite slurry is required to be zero [19].
(3) Funnel viscosity: the time required for 500 mL of slurry to flow out of a funnel. For bentonite slurry used in pipe-jacking construction, the funnel viscosity should be no less than 30 s.
(4) Apparent viscosity and plastic viscosity: These reflect the strength of the slurry’s network structure. When these values are within an appropriate range, the slurry exhibits a higher network structure strength.
(5) Gel strength and yield point: The minimum shear stress required to initiate flow in bentonite slurry after static conditions, overcoming internal friction, is defined as gel strength (initial and final gel strengths). The minimum shear stress required to maintain the slurry in a laminar flow state is referred to as the yield point.
(6) Yield point-to-plastic viscosity ratio: a key performance indicator for evaluating the shear-thinning behavior of slurry, reflecting the intensity of shear-thinning effects.

### 3.2. Test Materials

The selection of grouting materials is determined by the specific geological conditions of the project [11]. The circular pipe-jacking operation faces challenges such as high jacking resistance, ground collapse, and surface settlement. Conventional bentonite slurries often fail to meet construction requirements due to insufficient stability, poor lubricity, weak anti-seepage capacity, and limited formation adaptability [9].

To address these issues, this study proposed the incorporation of shell powder (a natural material) (Zhengzhou, China) into the conventional slurry mix [18]. The primary chemical component of shell powder is calcium carbonate (CaCO_3_), which participates in physical–chemical interactions within the slurry, promoting the formation of a dense and robust filter cake that reduces slurry permeation pathways. Additionally, its porous structure significantly enhances the slurry’s water retention and impermeability through physical adsorption. Sodium bentonite (Jiaxing, China), whose main mineral component is montmorillonite, undergoes volumetric expansion upon contact with water, forming a dense, low-permeability gel-like substance that effectively encapsulates and isolates the pipe segments from the gravel stratum. Soda ash (Mianyang, China), primarily composed of Na_2_CO_3_, acts as a critical activator by providing a strongly alkaline environment that displaces calcium ions between the bentonite mineral layers, thereby facilitating its thorough dispersion and hydration. Carboxymethyl cellulose (CMC) (Mianyang, China), an anionic cellulose ether, substantially increases the viscosity of the slurry through its efficient thickening, water retention, and suspension capabilities. Polyacrylamide (PAM) (Mianyang, China), a synthetic high-molecular-weight polymer, forms a lubricating layer at microscopic flow interfaces with its long-chain molecular structure, further reducing friction between the slurry itself and the pipe wall.

**Figure 1:**
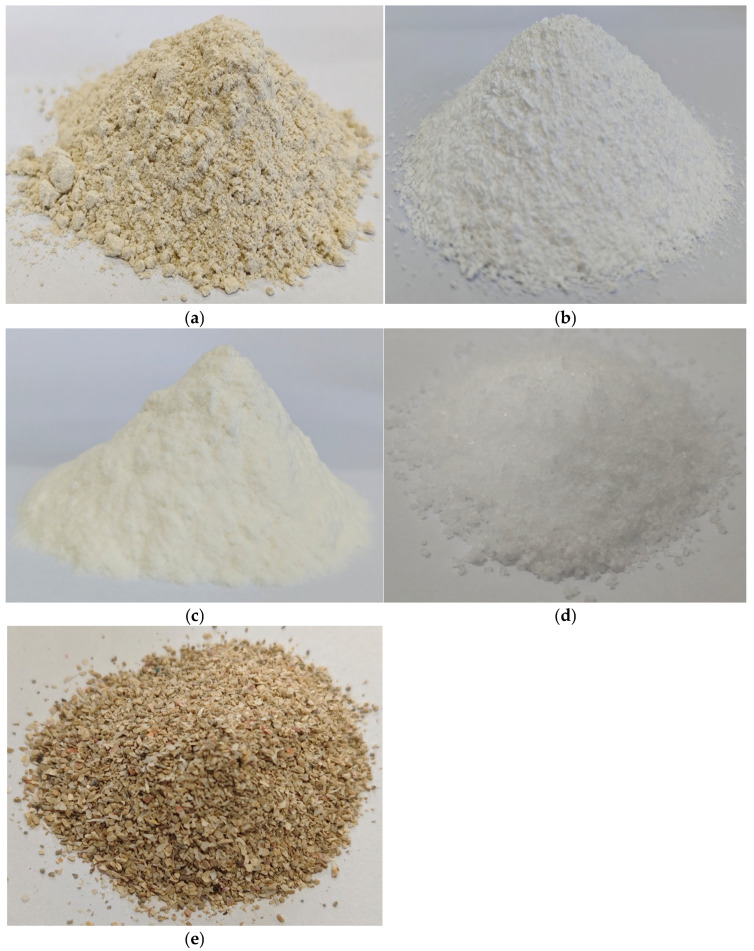
Bentonite (**a**), soda ash (**b**), CMC (**c**), PAM (**d**), and shell powder (**e**).

Based on comprehensive evaluation, sodium bentonite, soda ash, CMC, PAM, and shell powder were selected as raw materials for preparing the bentonite slurry. As illustrated in Figure 1, this mixture is expected to significantly enhance the stability, lubricity and supporting performance of the slurry, thereby effectively mitigating technical challenges associated with construction in gravel strata.

### 3.3. Test Methods

Orthogonal testing, an efficient, rapid, and economical experimental design method, can effectively reflect the outcomes of comprehensive testing through multi-factor and multi-level investigations [20]. Drawing on methods from reference [3], this study employs the first five columns of an L25(5^6^) orthogonal array to design the test scheme, systematically examining the influence of five factors (each with five levels) on the properties of bentonite slurry under different mix proportions. The five selected influencing factors are:

A. Mass percentage of bentonite relative to the total slurry mass;

B. Mass percentage of soda ash relative to the total slurry mass;

C. Mass percentage of CMC relative to the total slurry mass;

D. Mass percentage of PAM relative to the total slurry mass;

E. Mass percentage of shell powder relative to the total slurry mass.

The specific level values for each factor are listed in Table 1. The performance parameters of the bentonite slurry were measured according to the mix proportion combinations in the orthogonal test design table (Table 2). Table 2 presents the orthogonal test results. By calculating the mean and range of each factor, the importance of the influencing factors was ranked based on the fluctuation range of the slurry performance evaluation indicators and the optimal values for the corresponding content of each factor were determined. The combination of optimal levels for all factors was identified as the best mix proportion scheme from the orthogonal tests [3,21]. A total of 25 sets of tests were conducted to elucidate the influence mechanisms of component contents on key performance indicators of the bentonite slurry, thereby identifying the optimal mix proportion that meets engineering requirements [22].

### 3.4. Testing Procedure

**Figure 2:**
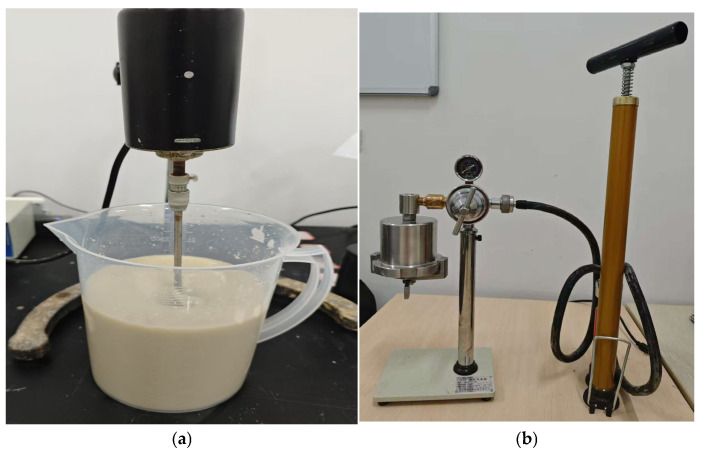

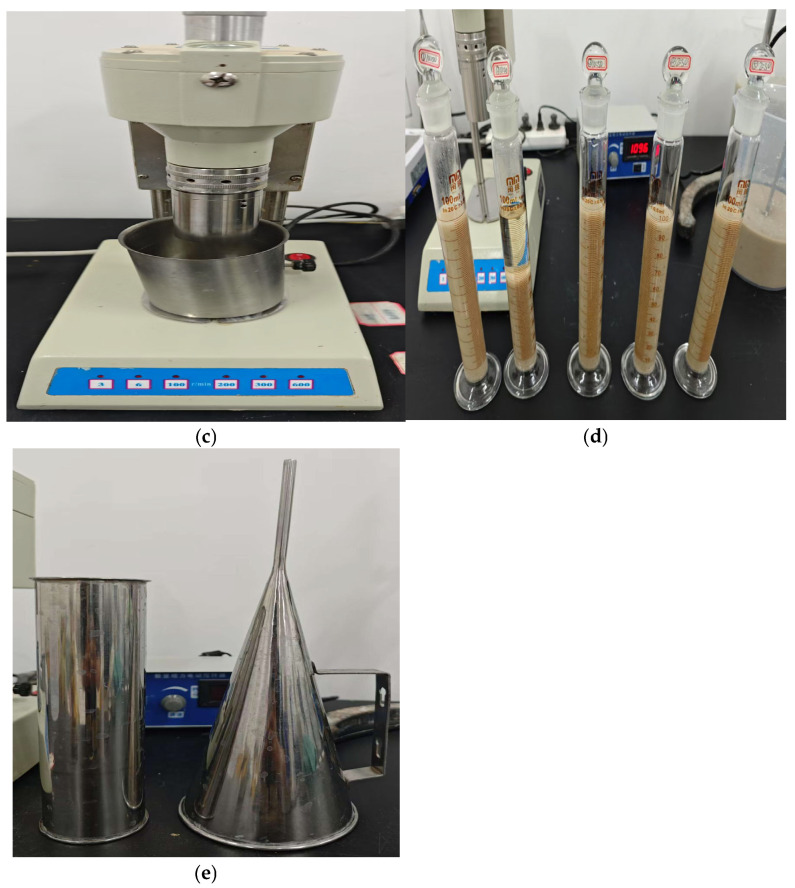
Instrument for measuring thixotropic mud properties ((**a**) low-speed mixer; (**b**) pneumatic filtration apparatus; (**c**) rotational viscometer; (**d**) cylinder; (**e**) funnel viscometer).

The performance parameters of the thixotropic slurry were tested using the apparatus shown in Figure 2. First, all raw material components were thoroughly mixed according to the designed proportions using a JJ-1A low-speed mixer (Figure 2a) (Changzhou Xiangtian Experimental Instrument Factory, Changzhou, China.). The mixture was stirred continuously at 800 r/min for 30 min to ensure that a homogeneous and stable slurry sample was obtained. Subsequently, parallel tests were conducted in four key aspects: (1) The filtration loss of the slurry under a pressure of 0.69 MPa over 30 min was measured using a ZNS-2A pneumatic filtration apparatus (Figure 2b) (Qingdao Shande Petroleum Instrument Co., Ltd., Qingdao, China.) to evaluate its wall-building capacity. (2) A ZNN-D6 six-speed rotational viscometer (Figure 2c) (Qingdao Shande Petroleum Instrument Co., Ltd., Qingdao, China.) was employed to determine viscosity values at different shear rates, from which rheological parameters such as plastic viscosity and yield stress were calculated. (3) To investigate the stability of the slurry, the prepared sample was left to stand in a 100 mL stoppered graduated cylinder (Figure 2d) for 24 h, and the water separation was recorded to assess its stability performance. (4) The workability of the slurry was tested using a standard ZMN-1 Marsh funnel viscometer (Figure 2e) (Qingdao Shande Petroleum Instrument Co., Ltd., Qingdao, China.) to obtain the funnel viscosity data. All tests were performed in a constant-temperature environment maintained at 25 ± 1 °C to ensure the accuracy and comparability of the experimental results. This comprehensive testing protocol covers the critical performance indicators of the thixotropic slurry, providing reliable experimental data for subsequent performance analysis and formulation optimization.

### 3.5. Analysis of Orthogonal Test Results on Mix Proportions

#### 3.5.1. Contribution of Influencing Factors to Key Slurry Performance Parameters

**Figure 3:**
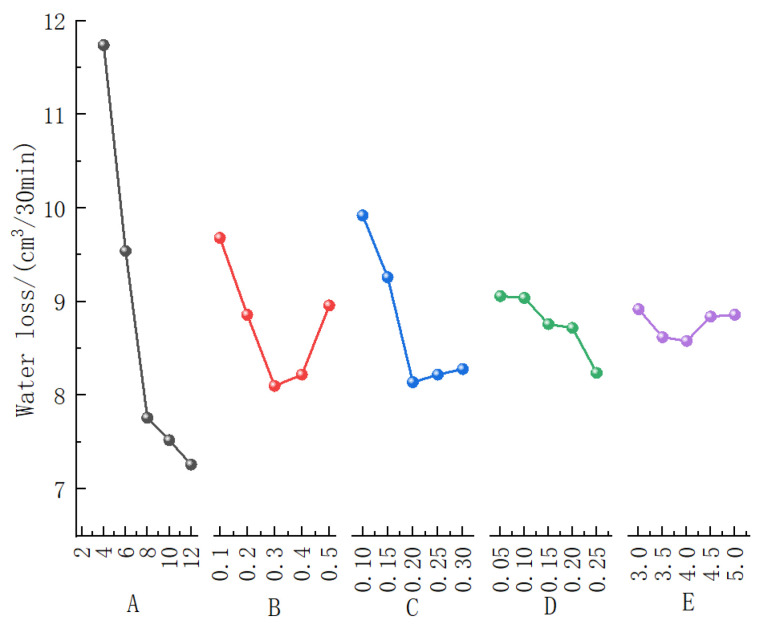
Contribution curve of five influencing factors to the water loss.

Figure 3 presents the variation curves of each factor’s influence on the water loss of the slurry. It can be observed that Factor A (bentonite content) has the most significant effect on water loss, followed by Factor C (CMC content), while the influences of Factor B (soda ash content) and Factor D (PAM content) are relatively minor. Factor E (shell powder content) exhibits the least influence. This conclusion is fully consistent with the order of importance of the influencing factors (A > C > B > D > E) obtained from the range analysis in Table 3.

Regarding the trend of water loss variation, as the bentonite content increases, the water loss shows a clear decreasing trend; however, when the content exceeds 8%, the rate of decrease tends to level off. Considering both performance improvement and economic efficiency, it is recommended to control the bentonite content at approximately 8%. For the effect of soda ash content, water loss initially decreases and then increases with increasing content, reaching an optimal value at a content of 0.3%. With an increase in CMC content, the water loss initially increases and then decreases. Taking economic cost into account, a CMC content of 0.2% is deemed most appropriate. The water loss reaches a minimum when the PAM content is 0.25%; therefore, a content of 0.25% is selected for Factor D. Similarly, the water loss is minimized when the shell powder content is 4%, leading to the selection of 4.0% for Factor E.

In summary, the optimal combination for minimizing water loss—a key performance indicator of the slurry—is: 8% bentonite content, 0.3% soda ash content, 0.2% CMC content, 0.25% PAM content, and 4% shell powder content. This corresponds to the optimal factor level combination: A_3_C_3_B_3_D_5_E_3_.

**Figure 4:**
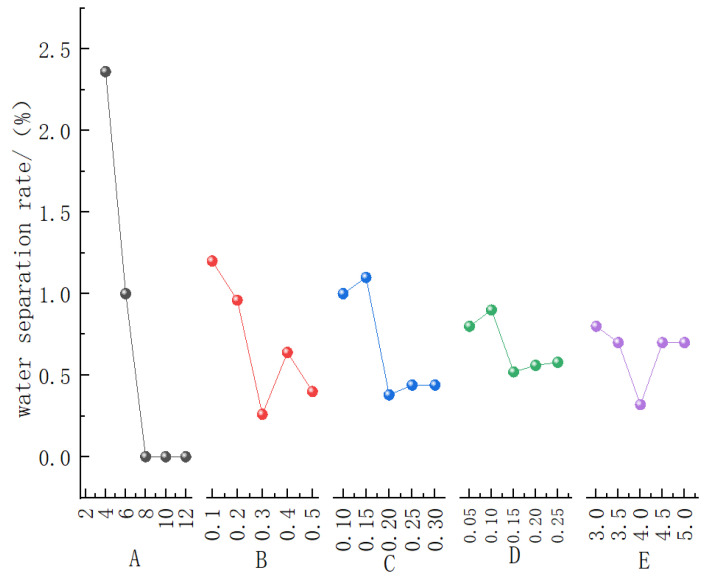
Contribution curve of five influencing factors to the water separation rate.

Figure 4 presents the contribution curves of the five influencing factors to the water separation rate, while Table 4 provides the range analysis results of the water separation rate for the bentonite slurry. It can be observed that Factor A (bentonite content) has the most significant influence on the water separation rate, followed in descending order by Factor B (soda ash content), Factor C (CMC content), Factor E (PAM content), and Factor D (shell powder content). This characteristic is fully consistent with the range analysis results shown in Table 4.

Regarding the variation trend of the water separation rate, when the bentonite content increases to 8%, the water separation rate can be reduced to zero. The water separation rate reaches its lowest value at a soda ash content of 0.3%, exhibiting a non-monotonic trend of initial decrease, followed by an increase, and then a subsequent decrease. After the CMC content increases to 0.2%, the water separation rate stabilizes. The water separation rate reaches a minimum at a PAM content of 0.15%, with its variation showing a fluctuating pattern. Meanwhile, the water separation rate is minimized at a shell powder content of 4%, demonstrating an initial decrease, followed by an increase, and then a slight decrease.

Based on the optimization results of all factors, the optimal mix proportion for minimizing the water separation rate is: A_3_ (8% bentonite), B_3_ (0.3% soda ash), C_3_ (0.2% CMC), E_3_ (0.15% PAM), and D_3_ (4% shell powder), i.e., the optimal combination is A_3_B_3_C_3_E_3_D_3_. This combination achieves the minimum water separation rate.

**Figure 5:**
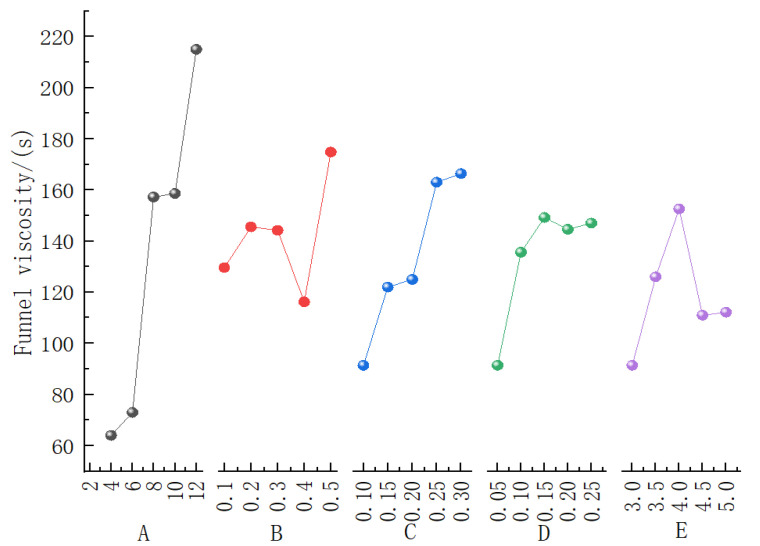
Contribution curve of five influencing factors to the funnel viscosity.

Figure 5 illustrates the contribution curves of the five influencing factors to the Marsh funnel viscosity, and Table 5 provides the corresponding range analysis results for the bentonite slurry. It is evident that Factor A (bentonite content) exerts the most significant influence on the funnel viscosity, followed by Factor C (CMC content) and Factor E (PAM content), while Factor B (soda ash content) and Factor D (shell powder content) exhibit relatively minor effects. This order of influence is consistent with the range analysis results presented in Table 5. A higher Marsh funnel viscosity indicates better slurry performance. Specifically, the funnel viscosity reaches its maximum at a bentonite content of 12%, an optimal value at a soda ash content of 0.5%, a peak at a CMC content of 0.3%, a maximum at a PAM content of 0.15% and the best performance at a shell powder content of 4%.

Therefore, to achieve the optimal Marsh funnel viscosity, the recommended mix proportion is: A_5_ (12% bentonite), B_5_ (0.5% soda ash), C_5_ (0.3% CMC), E_3_ (0.15% PAM), and D_3_ (4% shell powder), i.e., the optimal combination is A_5_B_5_C_5_E_3_D_3_.

#### 3.5.2. Influence of Raw Material Content on the Properties of Bentonite Slurry

**Figure 6:**
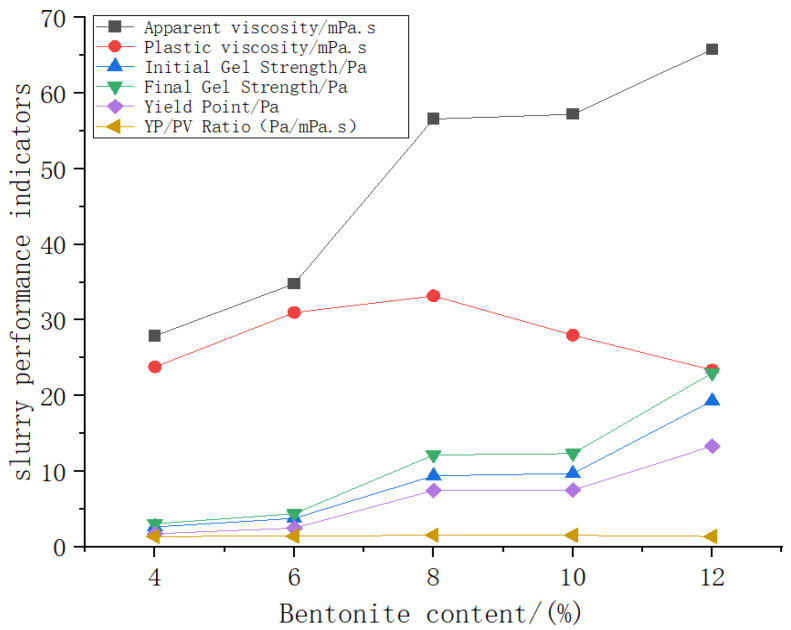
Trend curve of the impact of bentonite content on mud performance.

**Figure 7:**
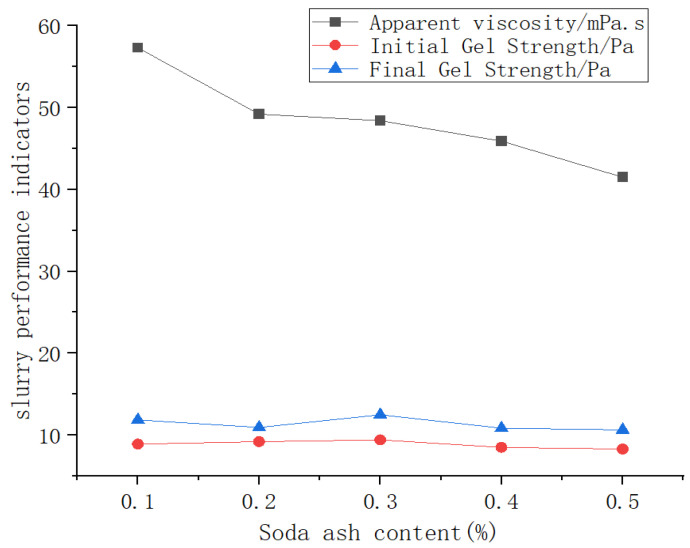
Trend curve of the impact of soda ash content on mud performance.

**Figure 8:**
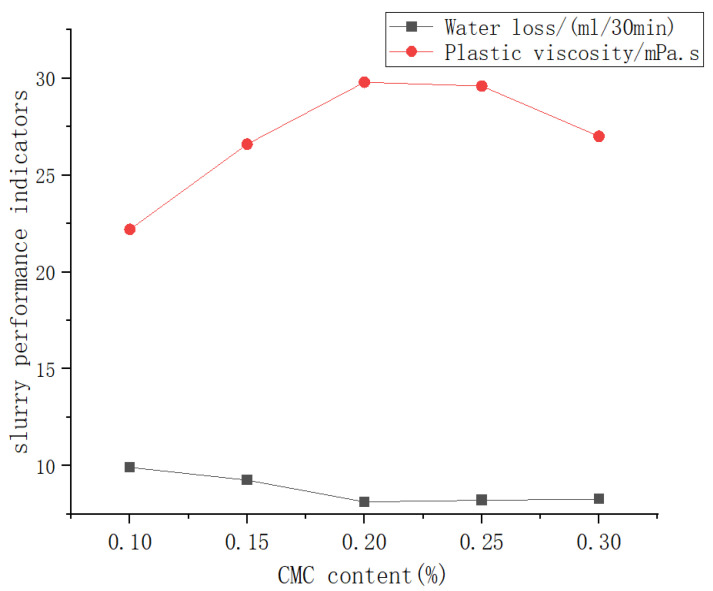
Trend curve of the impact of CMC content on mud performance.

**Figure 9:**
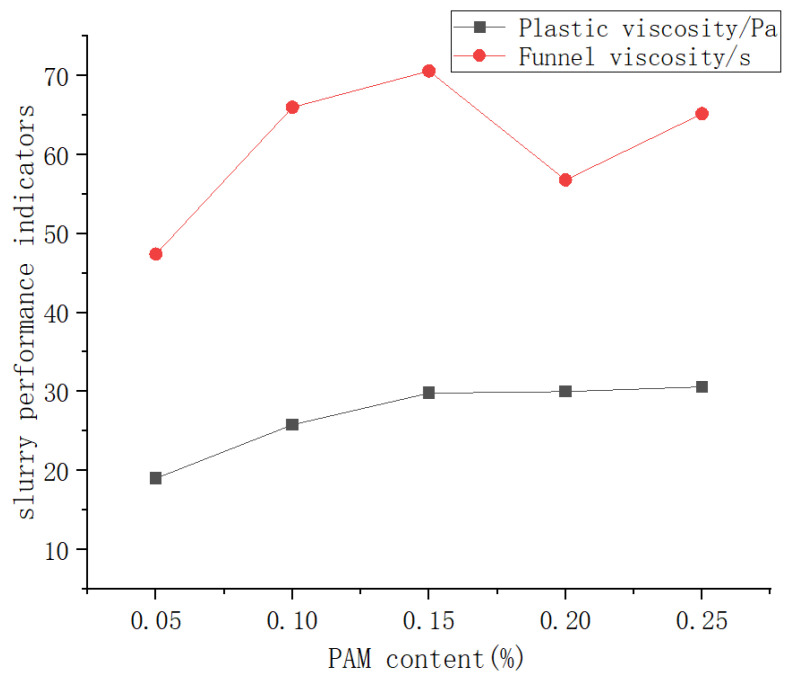
Trend curve of the impact of PAM content on mud performance.

**Figure 10:**
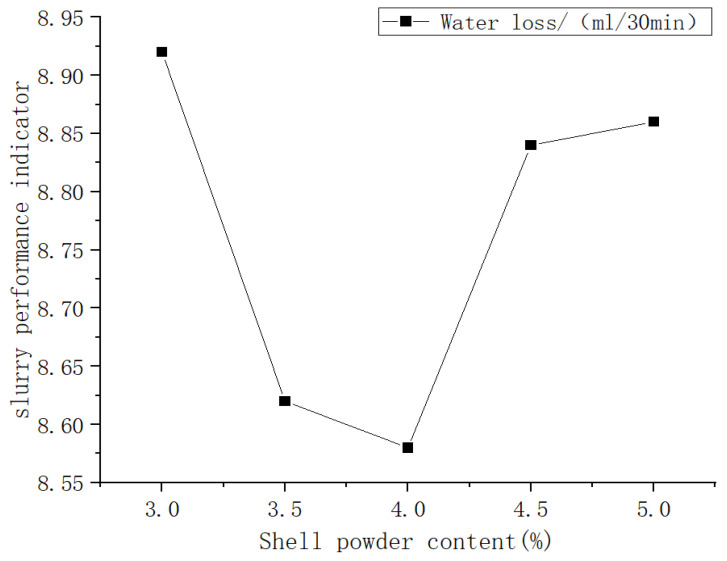
Trend curve of the impact of shell powder content on mud performance.

The influence of each constituent material’s content on various performance indicators of the slurry differs significantly. Based on the extent of this influence, Figure 6, Figure 7, Figure 8, Figure 9 and Figure 10 present and analyze the curves depicting how the content of different materials affects the corresponding performance indicators of the slurry.

Figure 6 illustrates the variation of slurry performance with bentonite content. As observed, the plastic viscosity reaches its maximum at a content of 8%, followed by a noticeable decrease at 10% and 12%, indicating that the network structure formed at 8% best facilitates thixotropic behavior. Meanwhile, the yield point, initial gel strength and final gel strength all exhibit a continuous increasing trend with higher bentonite content. Comprehensive analysis indicates that at a bentonite content of 8%, all key slurry parameters—including yield point-to-plastic viscosity ratio, apparent viscosity and gel strength values—fall within an ideal range. At this proportion, the slurry demonstrates favorable shear-thinning characteristics, excellent flowability, and strong structural stability, achieving an optimal balance between overall performance and structural strength.

Figure 7 shows the influence of soda ash content on slurry performance. With increasing soda ash content, the initial and final gel strengths first increase, then decrease and eventually stabilize, while the apparent viscosity shows a continuous decreasing trend. Notably, at a soda ash content of 0.3%, both initial and final gel strengths reach their peak values, and all performance parameters meet the target requirements. These results suggest that this specific content promotes the formation of an optimal network structure within the slurry, thereby achieving the best friction-reduction effect.

Figure 8 illustrates the variation of slurry performance with CMC content. It can be observed that the CMC content plays a significant regulatory role in slurry performance. As the CMC content increases, the plastic viscosity of the slurry first increases and then decreases nonlinearly, reaching a maximum at a content of 0.2%. This trend can be explained as follows: in the low content range (<0.2%), CMC molecules effectively enhance slurry viscosity by forming a stable three-dimensional network structure. However, when the content exceeds a critical value, the intermolecular interactions change, leading to a slight decline in viscosity performance. A similar trend is observed in the water loss behavior, with the optimal performance achieved at 0.2% CMC content, where water loss is minimized to meet the strictest engineering requirements. Although further increasing the CMC content causes a slight rebound in water loss, the magnitude of this effect is significantly reduced. A comprehensive analysis indicates that a 0.2% CMC addition achieves the best balance between plastic viscosity and water loss control while maintaining good structural strength, making it the most practically valuable optimized proportion for engineering applications.

Figure 9 shows the influence of the PAM content on slurry performance. The plastic viscosity of the bentonite slurry initially increases and then slightly decreases with increasing PAM content, peaking at a PAM content of 0.15%. The variation in Marsh funnel viscosity is more complex: it first increases, then decreases and increases again, reaching its maximum at a PAM content of 0.15%. This behavior may be attributed to the characteristics of PAM molecular chains. The long polymer chains can form a network structure within the slurry, increasing internal friction and significantly enhancing viscosity.

Figure 10 presents the effect of shell powder content on slurry performance. As the shell powder content increases, the water loss of the bentonite slurry first decreases and then increases, with the minimum water loss achieved at a content of 4%. This phenomenon stems from the excellent particle filling and dispersion properties of shell powder. At this optimal content, it effectively seals micropores in the slurry, significantly reducing filtrate loss.

Based on experimental measurements of various thixotropic slurry indicators, range analysis was employed to comprehensively evaluate the contribution of each factor to slurry performance and assess the influence patterns of component content on slurry performance metrics. The optimal thixotropic slurry formulation was ultimately determined as: 8% bentonite, 0.3% soda ash, 0.2% carboxymethyl cellulose (CMC), 0.15% polyacrylamide (PAM), 4% shell powder, and 87.35% water. The slurry prepared with this formulation demonstrates exceptional comprehensive performance, including significantly enhanced fluidity and dispersibility, minimal water loss, viscosity meeting standard requirements, the lowest water separation rate, and favorable thixotropic characteristics. The corresponding performance parameters are presented in Table 6.

## 4. Investigation of the Friction Reduction Effect of Bentonite Slurry

To more intuitively evaluate the friction reduction performance of bentonite slurry in concrete pipe jacking through gravel strata, designing and conducting indoor model tests is a simple and effective method [14]. Based on the previously determined optimal slurry mix proportion, three additional bentonite slurry mixes with different compositions were selected for comparison, with specific parameters provided in Table 7. The coefficient of friction between concrete specimens and gravel was measured under both grouted and non-grouted conditions to analyze the friction-reducing effect of the bentonite slurry.

**Figure 11:**
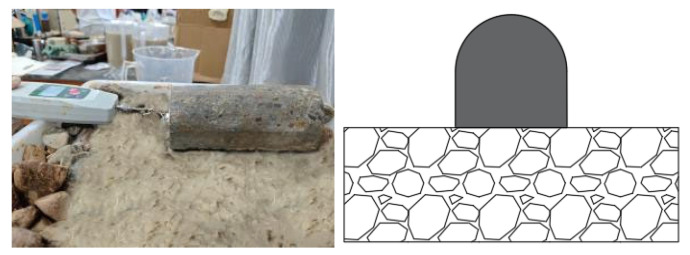
Flat-surface sliding drag-friction model test (**left**: actual diagram; **right**: ideal diagram).

**Figure 12:**
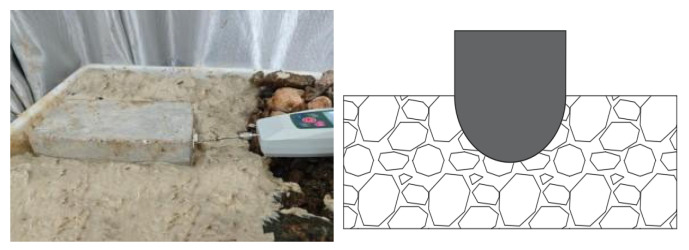
Curved-surface sliding drag-friction model test (**left**: actual diagram; **right**: ideal diagram).

A horseshoe-shaped concrete specimen, identical in material to the segments used at the construction site, was fabricated as shown in Figure 11. Two small-scale drag-friction model tests were designed and performed: one test involved placing the specimen with its flat surface facing downward to simulate the friction reduction between a flat concrete surface and gravel under the influence of bentonite slurry (hereinafter referred to as “flat surface sliding”), as shown in Figure 11; the other test placed the specimen with its curved surface facing downward to investigate friction reduction between an arched pipe-jacking surface and gravel with bentonite slurry (hereafter referred to as “curved surface sliding”), as illustrated in Figure 12.

To ensure the accuracy of the measured drag force in the drag-friction tests, each test was repeated 28 times under the same conditions, and the average value of the results was adopted. Table 8 presents the test results of the performance parameters of the bentonite slurry with the optimal mix proportion. Analysis of the experimental data indicates significant differences in the frictional characteristics at the interface between the concrete specimen and the gravel under different slurry mix proportions. Under the non-grouted reference condition, the average sliding friction forces for curved surface sliding and flat surface sliding were 17.35 N and 17 N, respectively, with corresponding friction coefficients of 0.430 and 0.422. These values provide a reliable baseline for evaluating the performance of different slurry mixes. When Mix 1 was used for grouting, the average sliding friction forces for curved surface sliding and flat surface sliding were 13.24 N and 13 N, respectively, with corresponding friction coefficients of 0.329 and 0.323. This represents a reduction of approximately 23.5% compared to the non-grouted baseline. Although a certain friction-reducing effect was observed, the insufficient viscosity due to the low bentonite content limited the lubrication performance. Mix 2 exhibited the optimal engineering performance. The average sliding friction forces for curved surface sliding and flat surface sliding were 11.18 N and 10.99 N, respectively, with corresponding friction coefficients of 0.277 and 0.272. This achieved a friction reduction rate of about 35.6%, attributable to the appropriate viscosity resulting from the optimal bentonite content. This viscosity effectively isolates the direct contact between the pipe and the soil while avoiding additional resistance caused by excessive viscosity. Although Mix 3 achieved a friction coefficient reduction of approximately 29.3%, the average sliding friction forces of 12.23 N and 12 N for curved surface sliding and flat surface sliding, respectively, along with noticeable subsidence of the specimen, indicate that while the increased bentonite content improved lubrication, it compromised the slurry’s support capacity, posing a risk of ground instability. Based on the comprehensive test data, Mix 2 achieves the best balance between friction reduction and ground stability, making it the recommended choice for engineering applications. However, in actual construction, dynamic adjustments should be made according to specific geological conditions and construction parameters to ensure optimal friction reduction while effectively maintaining face stability.

## 5. Results and Discussion

(1) Table 9 presents the materials and optimized mix ratios of thixotropic slurry used in different pipe-jacking projects in sandy cobble strata. It can be observed that for different pipe-jacking constructions, even when encountering similar sandy cobble strata, the selected thixotropic slurry materials and their mix ratios vary. Clearly, bentonite, soda ash, and CMC are the fundamental components of the thixotropic slurry. Based on these, additional materials such as polymers, polymeric additives, thickeners, or special admixtures have been incorporated for different projects.

Due to regional variability and differing geological conditions, the performance of bentonite slurry for long-distance pipe jacking in complex strata still requires continuous exploration and experimentation. This is essential for improving the study of parameter and mix-ratio optimization. Future research may focus on the development of new additive materials and their cost effectiveness to enhance the adaptability of slurry for pipe jacking in complex geological formations.

(2) The drag reduction performance of slurry with different mix ratios was evaluated through drag-friction model tests, which simulated both curved and planar sliding contact conditions. Given the inherent randomness and variability associated with laboratory model tests, a total of 28 repeated tests were conducted in this study. After excluding outliers, the average of the remaining data was taken as the final result, thereby effectively controlling random errors and improving the reliability of the measured friction coefficients. Meanwhile, certain limitations exist in the laboratory model tests: test conditions (such as the arrangement of pebbles, moisture state and stress environment) differ from those in actual engineering, and due to size constraints of the model, it is also difficult to fully capture the dynamic evolution of the slurry sleeve over time and space during long-distance pipe jacking. Future research may employ field pilot tests or numerical simulation methods to further verify and optimize the long term drag-reduction performance of slurry under complex geological conditions.

## 6. Conclusions

(1) Based on slurry mix proportion tests and multi-factor optimization analysis, the optimal slurry mix proportion was determined as: 8% bentonite, 0.3% soda ash, 0.2% CMC, 0.15% PAM, 4% shell powder, with the remainder being water (87.35%). The optimized slurry exhibited favorable fluidity and thixotropy, along with low water loss, achieving optimal performance across all measured indicators.

(2) Repeated indoor drag-friction model tests confirmed that the optimized thixotropic slurry (Mix Proportion 2) demonstrates effective friction reduction, decreasing the friction coefficient between the test block and gravel by 35.6%.

(3) The findings of this study provide important technical references for pipe-jacking construction under similar geological conditions. Given the substantial influence of slurry mix proportion on the quality and efficiency of pipe jacking, further research on slurry formulations remains necessary, particularly for gravel strata and other challenging ground conditions.

## Figures and Tables

**Table 1:** The specific level values of each factor.

Number of Levels	Factor				
	A%	B%	C%	D%	E%
1	4	0.1	0.1	0.05	3
2	6	0.2	0.15	0.1	3.5
3	8	0.3	0.2	0.15	4
4	10	0.4	0.25	0.2	4.5
5	12	0.5	0.3	0.25	5

**Table 2:** Orthogonal experimental design table and experimental results.

Experiment Number	A%	B%	C%	D%	E%	Water Loss (mL/30 min)	Funnel Viscosity/s	Water Separation Rate/%	Apparent Viscosity /mPa·s	Plastic Viscosity (PV) /mPa·s	Initial Gel Strength /Pa	Final Gel Strength /Pa	Yield Point	Yield Point/Plastic Viscosity Ratio
1	4	0.1	0.1	0.05	3	14.5	29	4	18.5	15	1.02	1.53	1.02	2.00
2	4	0.2	0.15	0.1	3.5	12.5	50	3.5	25	16	3.04	4.09	2.57	1.69
3	4	0.3	0.2	0.15	4	10.2	70	0.6	32	26	4.09	4.60	2.56	1.25
4	4	0.4	0.25	0.2	4.5	10.5	81.02	1.5	31.5	30	2.04	2.56	1.53	1.50
5	4	0.5	0.3	0.25	5	11	90	2.2	32.5	32	4.09	4.09	2.04	1.00
6	6	0.1	0.15	0.15	4.5	11	70	2	44.5	41	4.09	5.11	3.07	1.50
7	6	0.2	0.2	0.2	5	9.3	86.91	1.3	40	38	5.11	6.64	4.09	1.60
8	6	0.3	0.25	0.25	3	8	150	0.7	47	36	6.13	7.67	4.60	1.50
9	6	0.4	0.3	0.05	3.5	8.6	25	0	20	20	1.02	1.53	1.02	2.00
10	6	0.5	0.1	0.1	4	10.8	33	1	22.5	20	3.58	4.09	2.30	1.29
11	8	0.1	0.2	0.25	3.5	7.2	150	0	85	45	12.78	17.37	10.99	1.72
12	8	0.2	0.25	0.05	4	7.4	195	0	50	25	9.71	11.75	6.90	1.42
13	8	0.3	0.3	0.1	4.5	7.2	201	0	63	40	11.24	16.35	10.73	1.91
14	8	0.4	0.1	0.15	5	8.5	100	0	40	26	5.11	6.64	4.09	1.60
15	8	0.5	0.15	0.2	3	8.5	140	0	45	30	7.67	9.20	5.37	1.40
16	10	0.1	0.25	0.1	5	8.2	134	0	50	35	6.13	6.64	3.58	1.17
17	10	0.2	0.3	0.15	3	7.1	251	0	73	29	15.33	18.91	11.24	1.47
18	10	0.3	0.1	0.2	3.5	7.8	150	0	55	30	9.20	13.29	8.69	1.89
19	10	0.4	0.15	0.25	4	7	200	0	68	31	11.24	15.33	9.71	1.73
20	10	0.5	0.2	0.05	4.5	7.5	58	0	40	15	7.67	10.22	6.39	1.67
21	12	0.1	0.3	0.2	4	7.5	265	0	88.5	22	20.44	28.62	18.40	1.80
22	12	0.2	0.1	0.25	4.5	8	145	0	58	25	12.78	13.29	6.90	1.08
23	12	0.3	0.15	0.05	5	7.3	150	0	45	20	16.35	20.44	12.26	1.50
24	12	0.4	0.2	0.1	3	6.5	260	0	70	30	23.00	28.11	16.61	1.44
25	12	0.5	0.25	0.15	3.5	7	255	0	67.5	20	18.40	25.55	16.35	1.78

**Table 3:** Analysis of the water loss of thixotropic slurry.

Influencing Factors	Average Water Loss/(mL/30 min)					
	1	2	3	4	5	Range
A	11.74	9.54	7.76	7.52	7.26	4.48
B	9.68	8.86	8.1	8.22	8.96	1.58
C	9.92	9.26	8.14	8.22	8.28	1.78
D	9.06	9.04	8.76	8.72	8.24	0.82
E	8.92	8.62	8.58	8.84	8.86	0.34

**Table 4:** Results analysis of water separation rate of thixotropic slurry.

Influencing Factors	Average Water Separation Rate/(mL/30 min)					
	1	2	3	4	5	Range
A	2.36	1	0	0	0	2.36
B	1.2	0.96	0.26	0.64	0.4	0.94
C	1	1.1	0.38	0.44	0.44	0.72
D	0.8	0.9	0.52	0.56	0.58	0.38
E	0.8	0.7	0.32	0.7	0.7	0.48

**Table 5:** Analysis of the funnel viscosity of thixotropic slurry.

	Funnel Viscosity Average/(s)					
Influencing Factors	1	2	3	4	5	Range
A	64.0	72.98	157.2	158.6	215	151.0
B	129.6	145.58	144.2	116.2	174.8	58.6
C	91.4	122	124.98	163.0	166.4	75
D	91.4	135.6	149.2	144.59	147	57.8
E	91.4	126	152.6	111.0	112.18	61.2

**Table 6:** Thixotropic slurry performance parameters.

Overall Performance			Specific Parameters
Water Loss/(mL/30 min)			7
Viscosity/s			132
Water Separation Rate/%			0
Apparent Viscosity/mPa·s			45
Plastic Viscosity/mPa·s			28
Initial Gel Strength/Pa			5.11
Final Gel Strength/Pa			7.665

**Table 7:** Test material parameters.

Test Material	Specific Parameters (Bentonite:Soda Ash:CMC:PAM:Shell Powder)
Thixotropic slurries with different mix ratios	Mix Ratio1(6%:0.3%:0.2%:0.15%:4%)
	Mix Ratio2(8%:0.3%:0.2%:0.15%:4%)
	Mix Ratio3(10%:0.3%:0.2%:0.15%:4%)
C30 Concrete Test Cube	Dimensions: 20 cm (L) × 10 cm (W) × 5 cm (H) Radius of semicircular arc: 5 cm Mass: 4.11 kg

**Table 8:** Performance parameters of thixotropic slurry with optimal ratio.

	Grout Mix Ratio	Sliding Force/N	Friction Coefficient/μ
Curved surface sliding	Ungrouted	17.35	0.430
	Mix ratio 1 (grouted)	13.24	0.329
	Mix ratio 2 (grouted)	10.18	0.277
	Mix ratio 3 (grouted)	12.23	0.304
Flat surface sliding	Ungrouted	17.05	0.422
	Mix ratio 1 (grouted)	13.05	0.323
	Mix ratio 2 (grouted)	10.99	0.272
	Mix ratio 3 (grouted)	12.03	0.298

**Table 9:** Materials and optimized mix ratios of thixotropic slurry used in different pipe-jacking projects in sandy cobble strata.

Reference	Soil Layer Name	Materials and Optimal Mix Ratio
Zhao Ning [12]	Sandy Gravel	Bentonite:Soda Ash:Carboxymethyl Starch (CMS):PAM:Water = 80:0.15:0.3:0.15:1500
Liu Jie [23]	Cobble and Gravel	Bentonite:Soda Ash:CMC:Water = 1:0.06:0.0136:5.5
Zhou Haiwen et al. [24]	Water-rich Sandy Cobble	Bentonite:Sodium Carboxymethyl Cellulose (CMC-Na):PAM:Aluminum Sulfate = 7.18%:0.19%:0.2%:0.03%
Liu Ke et al. [10]	Water-rich Rounded Gravel	Bentonite:Soda Ash:CMC:Polymer Slurry Powder:Water = 10%:0.3%:0.2%:0.06%:89.44%
Wang Chunting [13]	Sandy Cobble	Bentonite:Soda Ash:PAM:Potassium Humate:Graphite Powder = 11%:5%:0.007%:1%:0.6%
Zhang Xue et al. [3,14]	Dry Sand Layer	Bentonite:Soda Ash:CMC:Water = 10%:0.5%:0.2%:89.3%
This Study	Water-rich Sandy Cobble	Bentonite:Soda Ash:CMC:PAM:Shell Powder:Water = 8%:0.3%:0.2%:0.15%:4%:87.35%

## Data Availability

The original contributions presented in this study are included in the article. Further inquiries can be directed to the corresponding author.

## References

[1] Hu R., Luo R., Ding W., Zhu B. (2024). Experimental study on supporting performance of thixotropic slurry and deposition characteristics of sand particles. J. Railw. Sci. Eng..

[2] Miao G., Huo Z., Chi L. (2024). Thixotropic characteristics of pipe-jacking slurry and formation mechanism of lubrication layer. Res. Urban Constr. Theory.

[3] Zhang X., Wan Z., Wang C., Liu Y., Liu S., Wang H., Zhang B. (2021). Ratio optimization and drag-reduction performance test of thixotropic slurry for rectangular pipe jacking in dry sandy strata. J. Eng. Geol..

[4] Wang Z., Xia Y., Yao Y., Zhao K. (2024). Performance study of drag-reducing slurry for long-distance pipe jacking in water-rich sandy strata. J. Liaoning Univ. Technol. (Nat. Sci. Ed.).

[5] Tang P. (2020). Application research on drag reduction technology for large-section rectangular pipe jacking: A case study of Suzhou utility tunnel. Bull. Geol. Sci. Technol..

[6] Feng R., Zhang P., Su S., Ma B. (2020). Grouting drag-reduction technology for large diameter and long-distance steel pipe jacking: Case study of Huangpu River upstream raw water conveyance project. Bull. Geol. Sci. Technol..

[7] Han T. (2025). Optimal ratio selection of thixotropic slurry for rectangular pipe jacking in clayey strata. J. Water Resour. Archit. Eng..

[8] Wang X., Li C., Liu Y. (2025). Optimization of slurry ratio and analysis of friction-reduction effect for long-distance rock pipe jacking. Sci. Technol. Eng..

[9] Liu L., Liu Y., Wang S. (2021). Void elimination method for over-excavation in rectangular pipe-jacked utility tunnels through sandy gravel stratum. China Water Wastewater.

[10] Liu K. (2024). Optimization of Lubricant Slurry Ratio for Pipe Jacking in Water-Rich Cobble Stratum. M.S. Thesis.

[11] Yan X. (2012). Application of Grouting Drag-Reduction Technology in Yellow River Pipe Jacking Project in Zhengzhou. Tunn. Constr..

[12] Zhao N. (2018). Construction control measures for long-distance pipe jacking through special gravel-cobble stratum. China Munic. Eng..

[13] Wang C., Long W. (2014). Experimental study on slurry formulation for large-diameter and long-distance pipe jacking engineering. J. Railw. Sci. Eng..

[14] Liu S., Zhang B., Zhang X., Fan D., Wang H., Yu M. (2022). Formulation optimization and performance analysis of the thixotropic slurry for large-section rectangular pipe jacking in anhydrous sand. Constr. Build. Mater..

[15] Wang Y., Liu N., Liang J., Xu H. (2024). Laboratory tests on the influence of thixotropic slurry composition on drag-reduction performance. Bull. Sci. Technol..

[16] Zeng J., Zhao D., Wang F., Hui H. (2024). Current status and development trends of pipe jacking tunneling technology. Mod. Tunn. Technol..

[17] Zhang S., Wei X., Wang X., Zhu H. (2021). Research status and prospect of pipe jacking resistance. Chin. J. Undergr. Space Eng..

[18] Xu H., Li H., Chi N., Liu C., Liu Z., Han P., Li H., Zhang Z. (2025). A Slurry for Supporting Borehole Walls in Cobble Stratum and Its Preparation Method..

[19] Wang M., Liu D. (2016). Test of Thixotropic Slurry Properties and Study of Resistance-Reducing Technology for Pipe Jacking Tunnel Construction. Mod. Tunneling Technol..

[20] Li Y., Hu C. (2010). Experimental Design and Data Processing.

[21] Wang Q., Zhang B., Wang H., Li Y., Hu Z., Lang B. (2020). Parameter optimization and stability analysis of lined underground gas storage clusters. J. Eng. Geol..

[22] Zhao X. (2006). Experimental Design Methods.

[23] Liu J. (2022). Construction control measures for large-diameter long-distance pipe jacking in gravel layers containing giant boulders. Constr. Mach. Maint..

[24] Zhou H., Zhou X., Ge L., Zhang F., Feng L.H., Liu Y. (2023). Optimization of mud ratio for freezing hole drilling in water-rich sand pebble formation. Sci. Technol. Eng..

